# TiO_2_-ZnO Binary Oxide Systems: Comprehensive Characterization and Tests of Photocatalytic Activity

**DOI:** 10.3390/ma11050841

**Published:** 2018-05-18

**Authors:** Katarzyna Siwińska-Stefańska, Adam Kubiak, Adam Piasecki, Joanna Goscianska, Grzegorz Nowaczyk, Stefan Jurga, Teofil Jesionowski

**Affiliations:** 1 Faculty of Chemical Technology, Institute of Chemical Technology and Engineering, Poznan University of Technology, Berdychowo 4, PL-60965 Poznan, Poland; adam.kubiak@outlook.com (A.K.); teofil.jesionowski@put.poznan.pl (T.J.); 2 Faculty of Mechanical Engineering and Management, Institute of Materials Science and Engineering, Poznan University of Technology, Jana Pawla II 24, PL-60965 Poznan, Poland; adam.piasecki@put.poznan.pl; 3 Faculty of Chemistry, Laboratory of Applied Chemistry, Adam Mickiewicz University in Poznan, Umultowska 89b, PL-61614 Poznan, Poland; asiagosc@amu.edu.pl; 4 NanoBioMedical Centre, Adam Mickiewicz University in Poznan, Umultowska 85, PL-61614 Poznan, Poland; nowag@amu.edu.pl (G.N.); stjurga@amu.edu.pl (S.J.)

**Keywords:** titanium dioxide, zinc oxide, binary oxide material, sol-gel method, organic dyes decomposition, photocatalysis

## Abstract

A series of TiO_2_-ZnO binary oxide systems with various molar ratios of TiO_2_ and ZnO were prepared using a sol-gel method. The influence of the molar ratio and temperature of calcination on the particle sizes, morphology, crystalline structure, surface composition, porous structure parameters, and thermal stability of the final hybrids was investigated. Additionally, to confirm the presence of characteristic surface groups of the material, Fourier transform infrared spectroscopy was applied. It was found that the crystalline structure, porous structure parameters, and thermal stability were determined by the molar ratio of TiO_2_ to ZnO and the calcination process for the most part. A key element of the study was an evaluation of the photocatalytic activity of the TiO_2_-ZnO hybrids with respect to the decomposition of C.I. Basic Blue 9, C.I. Basic Red 1, and C.I. Basic Violet 10 dyes. It was found that the TiO_2_-ZnO material obtained with a molar ratio of TiO_2_:ZnO = 9:1 and calcined at 600 °C demonstrates high photocatalytic activity in the degradation of the three organic dyes when compared with pristine TiO_2_. Moreover, an attempt was made to describe equilibrium aspects by applying the Langmuir-Hinsherlwood equation.

## 1. Introduction

Photocatalysis is an effective process for creating minerals out of pollutants in the air and water such as simple inorganic compounds in the presence of a catalyst [1]. The most common and widely described heterogeneous photocatalysts are transition metal oxides and semiconductors such as TiO_2_, ZnO, SnO_2_, and CeO_2_ [2,3,4,5]. Titanium dioxide is the most active of the compounds that have been tested. It is relatively cheap, photochemically stable, non-toxic, easily UV-activated, and insoluble in most reaction environments [6,7]. However, its application is limited because of its narrow photocatalytic region (α < 400 nm) and its ability to absorb only a small fraction (5%) of incident solar irradiation, which results from its relatively large band gap (anatase, ~3.2 eV) [8]. Many recent studies have focused on modifying the morphology and crystalline structure of TiO_2_ to improve its photocatalytic activity. Modification may be performed by adding transition metal ions (such as Cr, Zr, Mn, and Mo) [9,10,11,12], preparing a reduced form of TiO_2−x_, sensitization using dyes [13,14], doping with non-metals (such as N, S, C) [15,16,17], and using hybrid semiconductors such as TiO_2_-ZnO, TiO_2_-SiO_2_, etc. [18,19,20]. To increase the response of TiO_2_ to solar radiation, it is modified with ZnO, ZrO_2_, and SnO_2_ [21,22,23]. It has been proven that the formation of oxide hybrids is an appropriate tool for improving the photocatalytic ability of TiO_2_ materials. Selection of an appropriate modifier and its compatibility with the material are important for the hybrid’s physicochemical and optical properties. Each of the modifiers substantially affect the surface charge of the material and, therefore, enhance or weaken its photocatalytic capacity [24,25,26].

Zinc oxide is an attractive material due to its unique properties such as high chemical stability, high electrochemical coupling coefficient, high refractive index, high thermal conductivity, binding, antibacterial, and UV-protection properties [27]. Because of these properties, ZnO is added to materials and products including plastics, rubber, ceramics, paints, pigments, glass, cement, lubricants, ointments, adhesives, sealants, concrete, foods, batteries, ferrites, and fire retardants. Generally, zinc oxide occurs in nature in two main forms, which are hexagonal wurtzite and cubic zinc blende [28,29].

Titanium dioxide and zinc oxide have very similar physicochemical properties including nontoxicity, biocompatibility, thermal and chemical stability, insolubility in water, resistance to chemical breakdown and photo corrosion, and mechanical strength [30]. Many methods for the production of TiO_2_-ZnO oxide systems have been proposed using different precursors of titania and zinc oxide. These methods include an electrospinning technique [31], a chemical co-precipitation method [32], the sol-gel technique [33,34], or solvothermal and hydrothermal methods [35,36,37]. These methods enable precise control of the synthesis to obtain materials with useful properties. The process can be controlled by temperature changes, sequence and type of reagent dosage, rate of stirring, pH, the ratio of water to precursors, and calcination conditions. Depending on the process parameters, the products exhibit different physicochemical properties and structure. The physicochemical properties of TiO_2_-ZnO oxide systems depend on their morphology, the size of crystallites, and the crystallographic structure [31,32,33,34,35,36,37]. Moreover, ZnO has a band gap of 3.37 eV, which is slightly more negative than that of TiO_2_. Therefore, the synthesis of TiO_2_-ZnO oxide systems can result in the injection of conduction band electrons from ZnO to TiO_2_, which is favorable to electron-hole separation [38,39]. Therefore, the incorporation of these two materials into an integrated structure is of great significance because the resulting products may possess improved specific and well-defined physical and chemical properties, which were determined during their synthesis [31,32,33,34,35,36,37].

The aim of this work was to study the correlation between conditions of preparation (molar ratio of precursors, temperature of calcination) of TiO_2_-ZnO oxide systems and their properties including particle size distribution, morphology, crystalline and porous structure, and thermal stability. For the first time, this type of oxide material was used as a photocatalyst in the decomposition process of selected organic dyes (C.I. Basic Blue 9, C.I. Basic Red 1, and C.I. Basic Violet 10). In addition, theoretical description of performed photocatalytic process was presented. The Langmuir-Hinsherlwood equation and an assumption that pollutant decomposition is of pseudo-first-order reaction (PFO) were tested for this purpose.

## 2. Materials and Methods

### 2.1. Materials

Titanium(IV) isopropoxide (TTIP, 97%), C.I. Basic Blue 9 (Methylene Blue—MB, 95%), C.I. Basic Red 1 (Rhodamine 6G—R6G, 95%) and C.I. Basic Violet 10 (Rhodamine B—RhB, 95%) were purchased from Sigma-Aldrich. Zinc acetate dihydrate (99.5%), propan-2-ol (IPA, 99.5%), and ammonia (25%) were purchased from Chempur (Piekary, Śląskie, Poland). All reagents were of analytical grade and used without any further purification. The water used in all experiments was deionized.

### 2.2. Preparation of TiO_2_-ZnO Oxide Systems Using the Sol-Gel Method

**Figure 1:**
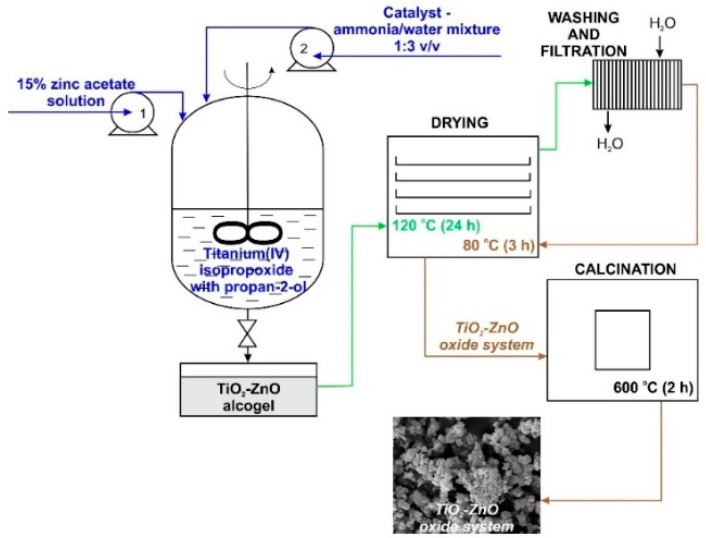
Synthesis of TiO_2_-ZnO binary oxide powders via the sol-gel method.

The synthesis of TiO_2_-ZnO oxide hybrids with TiO_2_:ZnO molar ratios of 9:1, 5:2, and 1:3 was performed by the sol-gel method. First, a reactor equipped with a T25 Basic type high-speed stirrer (IKA Werke GmbH, Staufen im Breisgau, Germany) was filled with a mixture containing an appropriate amount of TTIP in IPA. The resulting mixture was stirred at 1000 rpm. Afterward, an appropriate amount of 15% zinc acetate solution (the precursor of ZnO was dissolved in a mixture of IPA:H_2_O at a volume ratio of 1:3) was introduced at a constant rate of 5 cm^3^/min using an ISM833A peristaltic pump (ISMATEC, Wertheim, Germany). The synthesis was performed at room temperature. The reaction system was additionally stirred for 10 min. After this time, the promoter of hydrolysis (a mixture of ammonia and deionized water at a volume ratio of 1:3) was added at a constant rate of 1 cm^3^/min. The colloidal suspension was mixed for 1 h and the resulting alcogel was dried at 120 °C for 24 h. To remove impurities, the white precipitate was washed several times with deionized water. Lastly, the powder was dried at 80 °C for 3 h and additionally calcined at 600 °C for 2 h (Nabertherm P320 Controller, Lilienthal, Germany). The methodology of the synthesis of TiO_2_-ZnO oxide materials is presented in Figure 1.

### 2.3. Analysis of Materials

The particle sizes of the synthesized materials were measured by the non-invasive backscattering method (NIBS) using a Zetasizer Nano ZS (Malvern Instruments Ltd., Worcester, UK) instrument and enabling measurements in the diameter range 0.6–6000 nm. Each sample was prepared by dispersing 0.01 g of the tested product in 25 cm^3^ of propan-2-ol. The resulting system was sonicated for 15 min and then placed in a cuvette and analyzed.

The surface microstructure and morphology of the TiO_2_-ZnO binary oxide systems were examined on the basis of SEM images recorded from an EVO40 scanning electron microscope (Zeiss, Jena, Germany). Before testing, samples were coated with gold (Au) for 5 s using a Balzers PV205P (Oerlikon Balzers Coating SA,. Brügg, Switzerland) coater.

The crystalline structure of the synthesized binary oxide materials was analyzed by the X-ray diffraction method (XRD) using a D8 Advance diffractometer (Bruker, Karlsruhe, Germany) operating with Cu Kα radiation (α = 1.5418 Å), Ni filtered. The patterns were obtained in a step-scanning mode (Δ2θ = 0.05°) over an angular range of 10° to 80°.

High resolution transmission electron microscopy (HRTEM) images as well as dark field scanning TEM (DF STEM) selected area TEM diffractograms and EDS elemental maps were recorded by means of JEOL ARM 200F microscope (JEOL, Peabody, MA, USA) operating at an accelerating voltage of 200 kV. In order to prepare specimens, particular powders were dispersed in alcohol and then a few drops of such solution were placed on copper grids coated with carbon and formvar.

The surface composition of TiO_2_-ZnO oxide hybrids (content of TiO_2_ and ZnO) was analyzed by using energy dispersive X-ray spectroscopy (EDS) using a Princeton Gamma-Tech unit equipped with a prism digital spectrometer (Princeton Gamma-Tech, Princeton, NJ, USA). Representative parts of each sample (500 µm^2^) were analyzed to determine their actual surface composition.

The parameters of the porous structure of the obtained oxide powders were measured using a physisorption analyzer (ASAP 2020, Micromeritics Instrument Co., Norcross, GA, USA) operating based on a low-temperature adsorption of nitrogen. Before measurement, all materials were degassed at 120 °C for 4 h. Surface area was determined by the multipoint BET method using adsorption data in a relative pressure (*p/p_0_*) range of 0.05–0.30. The desorption isotherm was used to determine the pore size distribution based on the Barrett, Joyner, Halenda (BJH) model.

Characteristic functional groups present on the surface of the obtained materials were identified using Fourier transform infrared spectroscopy (FTIR). The measurements were performed using a Vertex 70 spectrophotometer (Bruker, Karlsruhe, Germany). Samples were prepared by mixing with KBr and pressing into small tablets. FTIR spectra were obtained in the transmission mode between 4000 cm^−1^ and 400 cm^−1^.

A thermogravimetric analyzer (Jupiter STA 449F3, Netzsch, Selb, Germany) was used to investigate the thermal stability of the synthesized materials. Measurements were carried out under nitrogen flow (10 cm^3^/min) at a heating rate of 10 °C/min over a temperature range of 30 °C to 1000 °C with an initial sample weight of approximately 5 mg.

### 2.4. Photocatalytic Tests

The photocatalytic activity of the obtained TiO_2_-ZnO binary oxide systems was evaluated based on the decomposition of C.I. Basic Blue 9 (MB), C.I. Basic Red 1 (R6G), and C.I. Basic Violet 10 (RhB) dyes (see, Table 1) in an initial concentration of 5 mg/dm^3^.

Photocatalysis was carried out in a laboratory reactor of UV-RS2 type (Heraeus, Hanau, Germany) equipped with a 150 W medium-pressure mercury lamp as a UV light source surrounded by a water-cooling quartz jacket. First, an appropriate amount of photocatalyst (TiO_2_-ZnO binary oxide material) was added to a glass tube reactor containing 100 cm^3^ of the model organic impurity. The suspension was stirred using an R05 IKAMAG magnetic stirrer (IKA Werke GmbH, Staufen im Breisgau, Germany) for 30 min in darkness to determine the adsorption/desorption equilibrium. After this time, the radiation was turned on to initiate the photocatalytic reaction. The process was carried out for a maximum of 150 min. In the next step, the irradiated mixtures were collected from the reactor at regular intervals and centrifuged to separate the photocatalyst. The concentration of C.I. Basic Blue 9, C.I. Basic Red 1, or C.I. Basic Violet 10 (after adsorption and UV irradiation) was analyzed using a UV-Vis spectrophotometer (V-750, Jasco, Oklahoma City, OK, USA) at a wavelength of 664 nm (for MB), 526 nm (for R6G), or 553 nm (for RhB) using water as a reference. The photocatalytic activity of the TiO_2_-ZnO binary oxide systems was determined by calculating the yield of dye degradation (*W*) using the formula below.

(1) $$W(\%)=\left(1-\frac{C_{t}}{C_{0}}\right)\cdot 100\%$$

where *C*_0_ and *C_t_* are the concentrations of the dye prior to and after irradiation, respectively.

### 2.5. Kinetic Study

Kinetic energy of the photocatalytic decomposition of selected organic dyes was described based on the Langmuir-Hinsherlwood equation [40] assuming that pollutant decomposition is of a pseudo-first-order reaction nature. The equation presents dependence between the dye concentration in the aqueous vs. time of UV irradiation.

(2) $$r=\frac{dC}{dt}=k\left(\frac{KC}{1+KC}\right)$$

Assuming that the degradation process of the dye is of pseudo-first-order reaction nature, the constant reaction rate can be determined as the slope of the linear regression.

(3) $$-\ln\left(\frac{C_{t}}{C_{0}}\right)=kt$$

where *k* is the degradation rate of organic dye, min^−1^, *K* is the equilibrium constant of adsorption of the dye on the surface of the catalyst, *C*_0_, *C_t_* are concentrations of the dye compound in aqueous solution before irradiation (*t* = 0) and after define time *t*.

Estimation of constant reaction rate *k* enables determination of the half-life time of the model organic pollutant.

(4) $$t_{\frac{1}{2}}=\frac{\ln2}{k}$$

## 3. Results and Discussion

### 3.1. Dispersive and Morphological Characteristics

The results of dispersive analysis (see Table 2) show that both synthetic TiO_2_ and ZnO (without thermal treatment) have monomodal particle size distributions. The TiO_2_ and ZnO samples (denoted Ti and Zn) contain particles in the diameter ranges of 531–1720 nm and 220–615 nm, respectively. Dispersive analysis of the synthetic TiO_2_-ZnO oxide systems showed that the molar ratio of the precursors significantly affects the particle sizes of the resulting materials. Samples obtained with TiO_2_:ZnO molar ratios of 9:1; 5:2, and 1:3 denoted as Ti9Zn1, Ti5Zn2, and Ti1Zn3, which contain particles in the ranges 459 nm to 1110 nm, 459 nm to 1480 nm, and 396 nm to 825 nm, respectively. The results show that products with smaller particles were obtained when a higher content of ZnO was used.

It was also confirmed that increasing the temperature of calcination leads to the production of products with larger particles as a result of sintering and agglomerate formation. This situation was observed for all of the oxide materials except samples Ti5Zn2_600 and Ti1Zn3_600. All calcined TiO_2_-ZnO oxide systems exhibit monomodal particle size distributions. Synthetic oxide systems obtained with different TiO_2_:ZnO molar ratios (samples Ti9Zn1_600, Ti5Zn2_600, and Ti1Zn3_600) contain particles in the diameter ranges 531 nm to 1280 nm, 459 nm to 955 nm, and 255 nm to 615 nm, respectively. Among the calcined samples, those obtained with the highest molar contribution of ZnO were composed of the smallest particles.

**Figure 2:**
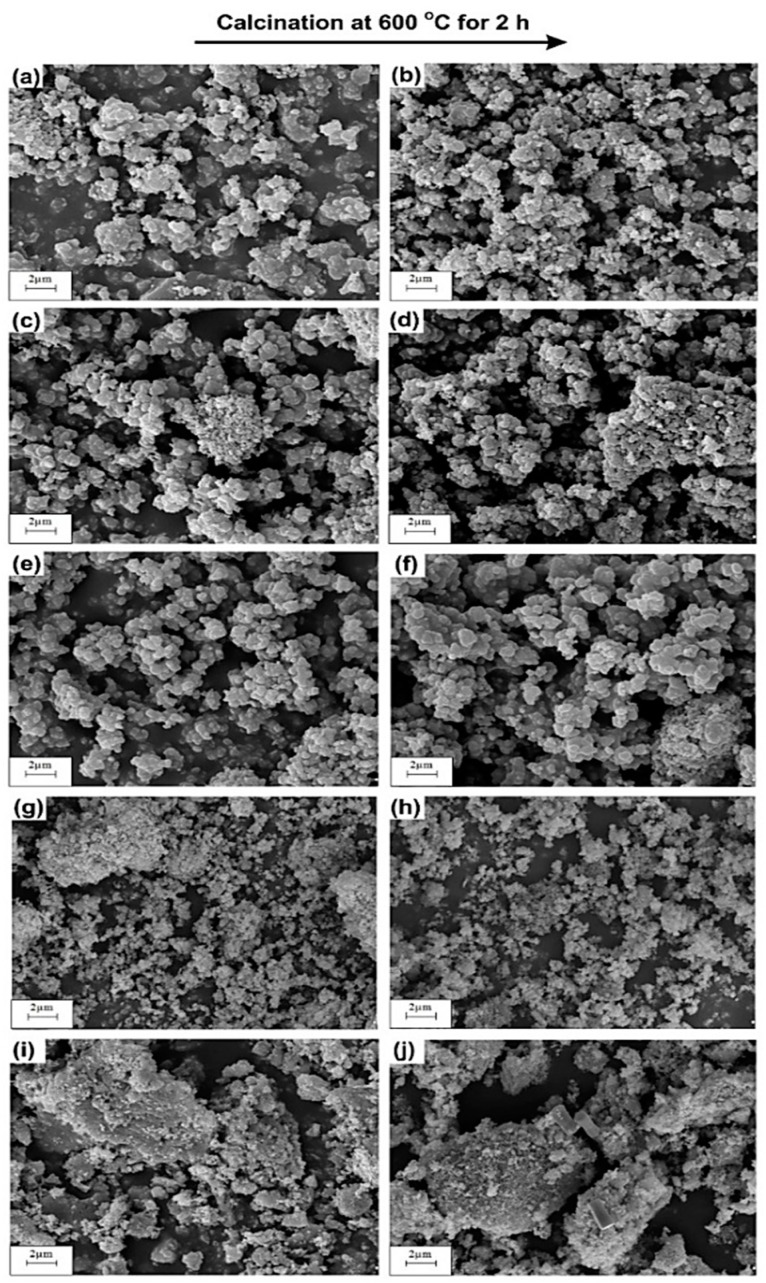
SEM images of obtained samples: (**a**) Ti; (**b**) Ti_600; (**c**) Ti9Zn1; (**d**) Ti9Zn1_600; (**e**) Ti5Zn2; (**f**) Ti5Zn2_600; (**g**) Ti1Zn3; (**h**) Ti1Zn3_600; (**i**) Zn and (**j**) Zn_600.

The SEM microphotographs of TiO_2_ and ZnO samples (uncalcined and calcined at 600 °C, Figure 2a,b,i,) show the presence of particles of almost spherical shape with high homogeneity. Moreover, the SEM micrographs for all analyzed oxide samples confirm the presence of particles, which exhibit a high tendency towards agglomeration. SEM observations of the synthesized TiO_2_-ZnO oxide systems (see Figure 2c–h) show that the molar ratio of the precursors does not have any significant effect on the morphology of the resulting systems. The SEM microphotographs of the studied samples confirm the presence of particles with precisely designed diameters, which corresponds to those indicated in the particle size distributions. Wang et al. [41] who synthesized a TiO_2_-ZnO oxide system through a sol-gel method using ammonia as a catalyst obtained results analogous to those reported here. Tsai et al. [42] noted that the TiO_2_-ZnO oxide system contains particles of a spherical shape, which show a high tendency towards agglomeration. Similarly, Ullah et al. [43] demonstrated that a TiO_2_-ZnO oxide system synthesized via a sol-gel method using dimethylaminoethanol was composed of particles of spherical shape with a high tendency to agglomerate.

### 3.2. Structural Characteristics

**Figure 3:**
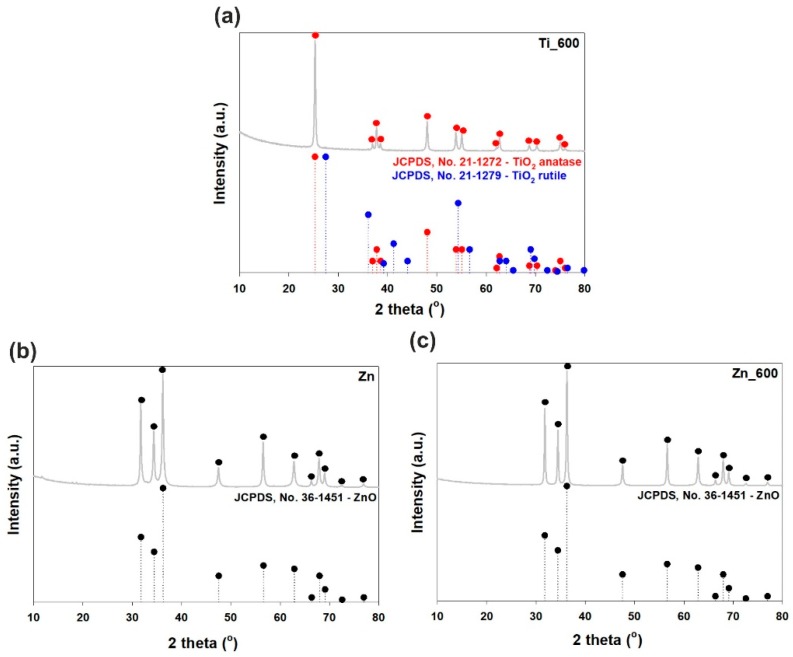
WAXS patterns of (**a**) calcined TiO_2_; (**b**) uncalcined ZnO; and (**c**) calcined ZnO.

The XRD pattern of titanium dioxide calcined at 600 °C (see Figure 3a) shows a strong diffraction peak at 2θ = 25.2, which corresponds to the anatase structure (JCPDS (Joint Committee on Powder Diffraction Standards), No. 21-1272). Less intense, characteristic diffraction peaks found at 36.95°, 37.8°, 38.58°, 48.05°, 53.89°, 55.06°, 62.12°, 62.69°, 68.76°, 70.31°, 75.03°, and 76.02° are also strictly related to the anatase phase. The rutile TiO_2_ phase is not detected in this sample. Moreover, the XRD patterns of un-calcined zinc oxide and zinc oxide calcined at 600 °C (see Figure 3b,c) correspond to the wurtzite phase of ZnO with high intensity peaks located at 31.77°, 34.42°, and 36.25° (JCPDS No. 36-1451). These results prove that thermal treatment does not change the crystalline structure of the zinc oxide materials.

**Figure 4:**
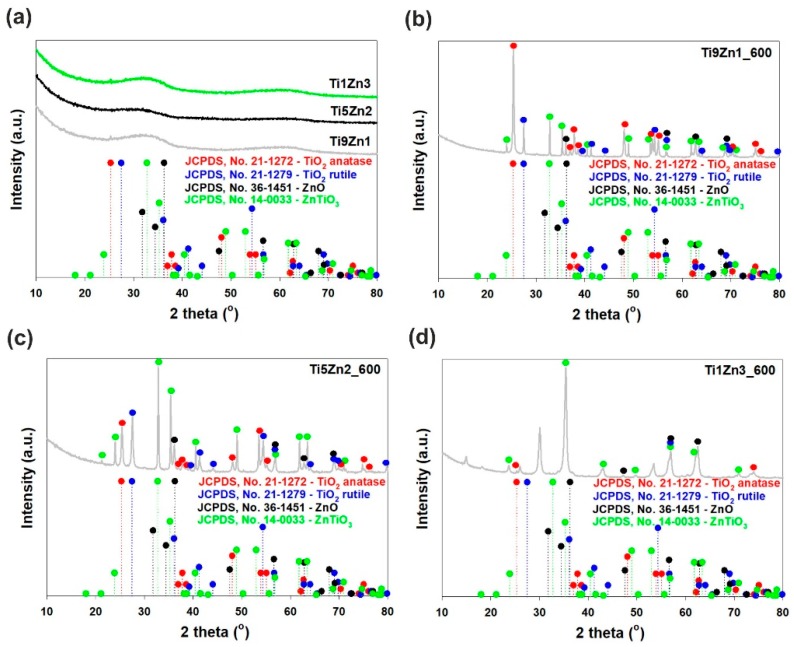
WAXS patterns of TiO_2_-ZnO oxide systems: (**a**) synthesized with different molar ratios without thermal treatment and prepared with TiO_2_:ZnO molar ratios of (**b**) 9:1; (**c**) 5:2; (**d**) 1:3, and subjected to thermal treatment.

The XRD patterns of the synthetic, un-calcined TiO_2_-ZnO oxide hybrids (see Figure 4a) do not show diffraction peaks of the TiO_2_ and ZnO phases. The obtained samples are amorphous. It has been reported that the combination of titania with zinc oxide leads to inhibition of the phase formation of the ZnO crystalline structure. These obtained results suggest that some Zn^2+^ cations can incorporate into the titania network [44], which follows from the fact that the ionic radii of Zn^2+^ (ca. 60 pm) and Ti^4+^ (ca. 60.5 pm) are similar [45]. The XRD pattern of sample Ti9Zn1_600 (obtained with a TiO_2_:ZnO molar ratio of 9:1 and calcined at 600 °C) confirms the formation of a crystalline material containing both titania and zinc oxide phases (see Figure 4b). Our results are in agreement with those of Stroyanova, Shalaby, and Moriadi [21,46,47]. Anatase was observed to be the dominant phase in sample Ti9Zn1_600. Characteristic diffraction peaks found at 25.28°, 36.95°, 37.8°, 38.58°, 48.05°, 53.89°, 55.06°, 70.31°, 75.03° and 76.02° were attributed to the anatase phase. For this sample, the peaks located at 27.45°, 39.19°, 41.23°, 44.05°, 54.32°, 56.64°, 65.48°, 69.01°, 69.79° and 79.82° correspond to the rutile phase. The XRD pattern of the obtained material also exhibited characteristic peaks with low intensity observed at 2θ = 36.25°, 56.6°, 62.86°, and 67.96°, which are characteristic for the ZnO structure. Additionally, the reflections observed at 2θ = 23.86°, 32.73°, 35.25°, 41.52°, 48.92°, 52.96°, 56.79°, 61.70°, 63.40°, 68.72°, and 70.91° can be identified with a cubic ZnTiO_3_ phase (JCPDS No. 14-0033). For sample Ti5Zn2_600 (obtained with a TiO_2_:ZnO molar ratio of 5:2 and calcined at 600 °C; Figure 4c) with increasing content of ZnO, the characteristic peaks of anatase, and rutile TiO_2_ gradually decreased [48]. Moreover, characteristic diffraction peaks found at 36.25°, 56.6°, 62.86°, and 67.96° are strictly related to the ZnO phase. In Ti9Zn1_600 and Ti5Zn2_600 samples crystallization of photoactive ZnTiO_3_, which is the result of reaction between titania and zinc oxide was observed. For the analyzed materials, the intensity of the ZnTiO_3_ peaks also increased. The XRD pattern of the TiO_2_-ZnO oxide system obtained with a TiO_2_:ZnO molar ratio of 1:3 and calcined at 600 °C (sample Ti1Zn3_600, Figure 4d) contained diffraction signals at 2θ values of 23.86°, 35.25° 43.10°, 48.92°, 56.79°, 61.70° and 70.91°, which is characteristic for the ZnTiO_3_ structure. We also observed that increasing the molar ratio of ZnO until 3 leads to formation of photoinactive Zn_2_TiO_4_. The results obtained here are identical to those reported by other researchers [48,49,50,51].

**Figure 5:**
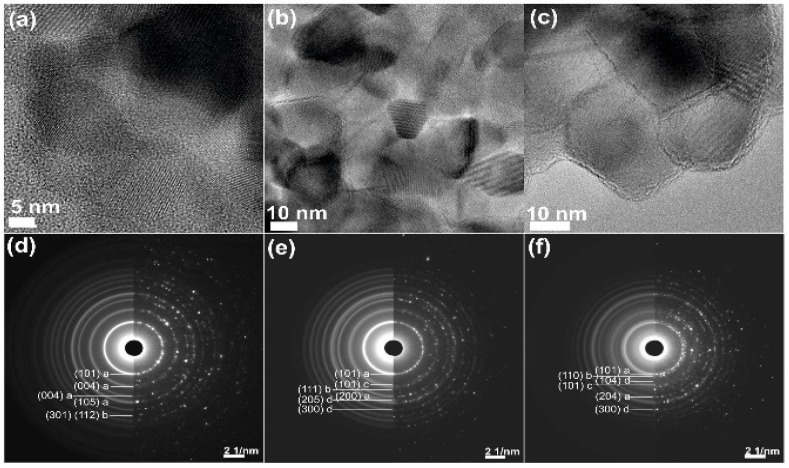
HRTEM images of samples: (**a**) Ti_600; (**b**) Ti9Zn1_600; and (**c**) Ti5Zn2_600, SATEM diffractograms of samples: (**d**) Ti_600; (**e**) Ti9Zn1_600; and (**f**) Ti5Zn2_600. Additionally, hkl planes of individual crystalline phases were denoted as: a—anatase, b—rutile, c—ZnO, d—ZnTiO_3_.

HRTEM measurements confirmed that all prepared materials exhibit highly crystalline forms (see Figure 5a–c).

In order to confirm the crystalline structure of studied samples in the selected area, TEM diffraction experiments were conducted. The obtained results clearly confirmed high crystallinity of investigated samples and proved that crystallinity of TiO_2_-ZnO is quite complex. However, the TEM diffractograms of the same high resolution as XRD results show well-distinguishing diffraction rings, which corresponds to data from XRD. The results of SATEM diffraction are presented in Figure 5d–f. The diffractograms were analyzed using CHT Diffraction Analysis [52]. The most distinctive rings of diffraction were indexed and compared to XRD data.

**Figure 6:**
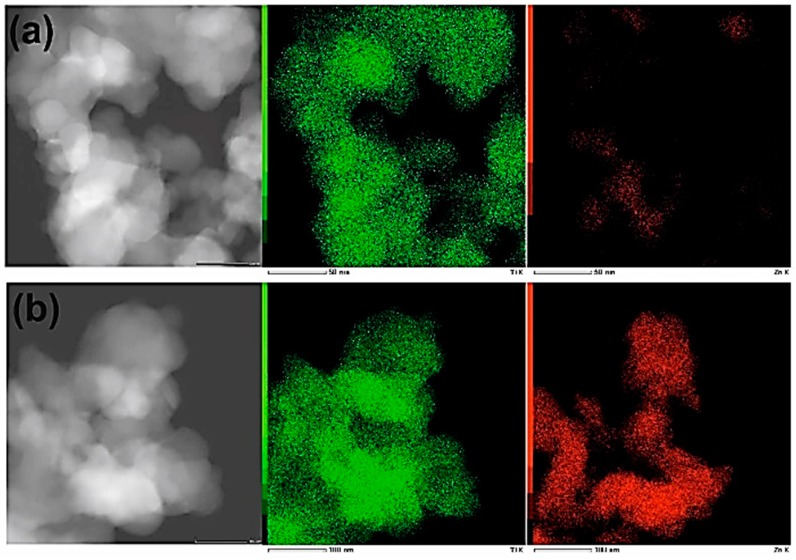
DFSTEM images and elemental maps of samples: (**a**) Ti9Zn1_600 and (**b**) Ti5Zn2_600. Ti and Zn are indicated as a green and a red color, respectively.

EDS (energy dispersive spectroscopy) experiments were carried out to verify the distribution of materials’ components (elements) within the samples. The results (see Figure 6) indicate that distribution of Zn is not uniform.

### 3.3. Surface Composition

**Figure 7:**
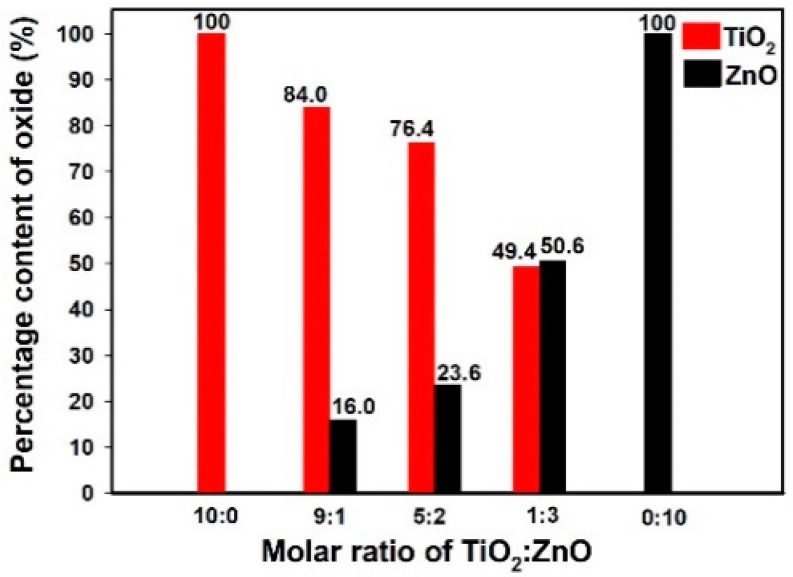
The percentage content of titania and zinc oxide in the calcined materials.

Figure 7 presents the percentage content of titanium oxide and zinc oxide in the analyzed oxide systems. The results confirmed the efficiency of the sol-gel route of synthesis. Moreover, energy dispersive X-ray microanalysis showed that changing the molar ratio of the initial precursors affects the content of the corresponding oxides in the structure of the final materials. As was expected, the highest quantity of titania (84.0%) was observed in sample Ti9Zn1_600 (obtained with the molar ratio TiO_2_:ZnO = 9:1) and the highest quantity of zinc oxide (50.6%) in sample Ti1Zn3_600. It was concluded that the sol-gel method makes it possible to obtain materials with strictly defined properties whose composition is mainly determined by the molar ratio of the initial precursors.

### 3.4. Porous Structure Parameters

The surface area of any material is the most important factor for influencing its catalytic activity. The results of textural characteristics of the obtained materials are summarized in Table 3. Samples of TiO_2_-ZnO oxide systems that were not subjected to thermal treatment exhibit a relatively high surface area. The highest value, A_BET_ = 494.7 m^2^/g, was observed for sample Ti9Zn1, which may be directly related to the dispersive nature of the analyzed material. This sample contained particles with smaller diameters than those of pure TiO_2_, which is directly linked to the porous structure parameters of the products of synthesis. Slightly poorer porous structure parameters were observed for pure TiO_2_ and sample Ti5Zn2 (with a molar ratio of TiO_2_:ZnO = 5:2), which had surface areas (A_BET_) of 488.6 m^2^/g and 475.8 m^2^/g. Moreover, an increase in the molar contribution of zinc oxide in the final product caused a significant decrease in the specific surface area, which was measured at 97.0 m^2^/g for sample Ti1Zn3 (with a molar ratio of TiO_2_:ZnO = 1:3) and 27.2 m^2^/g for ZnO. Our observations align with those of Prasannalakshmi and Shanmugam [51].

The samples that had undergone calcination exhibited a large decrease in the surface area. The highest surface area (7.6 m^2^/g) for these TiO_2_-ZnO oxide materials was recorded for sample Ti9Zn1_600. Thermal treatment also led to a significant decrease in the pore volume and a slight increase in the pore diameters of the obtained materials. The calculated values also imply that the surface area decreases with increased ZnO content.

### 3.5. FTIR Analysis

**Figure 8:**
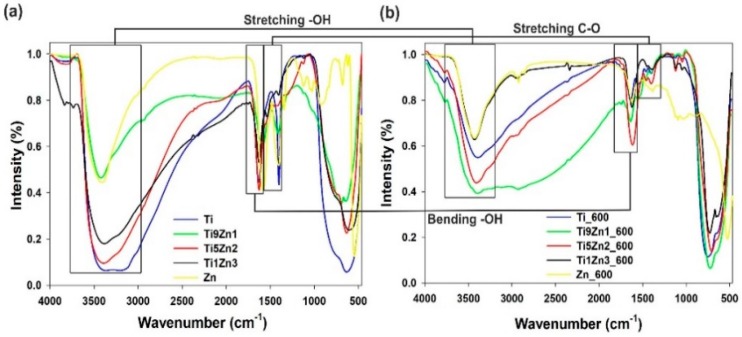
FTIR spectra of (**a**) un-calcined and (**b**) calcined TiO_2_, ZnO, and TiO_2_-ZnO samples.

The FTIR spectra of TiO_2_-ZnO binary oxide materials (see Figure 8) show absorption peaks at 550 cm^−1^ and 650 cm^−1^ ascribed to symmetric stretching vibrations of ≡Ti−O−Ti≡ and the vibration mode of −Zn−O−Ti≡ groups [47,50,53]. The band at 1400 cm^−1^ indicates stretching vibrations of C–O bonds [54]. Moreover, the FTIR spectra of the synthetic TiO_2_-ZnO oxide systems contain absorption peaks at 3440 cm^−1^ and 1630 cm^−1^, which is attributed to physically adsorbed water (–OH) and N–H stretching vibrations [55,56,57].

The FTIR spectra of titanium dioxide (uncalcined and calcined) show three characteristic bands at 550 cm^−1^, 1400 cm^−1^, and 3400 cm^−1^, which is associated respectively with stretching vibrations of ≡Ti−O, C–O, and –OH bonds. Analysis of the FTIR spectra of zinc oxide reveals a peak characteristic for zinc oxide (Zn–O) at 500 cm^−1^. The broad absorption peak appearing at 700 cm^−1^ to 1100 cm^−1^ is characteristic for non-reacted products such as CH_3_COO^−^ and NH_4_^+^. Moreover, the peak at approximately 3400 cm^−1^ is ascribed to stretching vibrations of O–H bonds, which are indirectly related to water physically adsorbed on the surface. The FTIR results for synthetic TiO_2_-ZnO oxide hybrids showed absorption peaks for ≡Ti−O−Ti≡ bonds at 550 cm^−1^, Zn−Ti−O bonds at 650 cm^−1^, and –OH groups at 3400 cm^−1^. Analysis of the FTIR spectra for samples Ti9Zn1, Ti5Zn2, and Ti1Zn3 reveals significant changes in the intensities of the relevant bands, which depend on the molar ratio of the precursors. Moreover, for TiO_2_-ZnO oxide systems calcined at 600 °C (Ti9Zn1_600, Ti5Zn2_600, Ti1Zn3_600), a decrease in the intensity of the –OH peak at 3400 cm^−1^ was observed. The spectra show that the intensity of the absorption bands around 650 cm^−1^, which correspond to ≡Ti−O−Ti≡ bonds, increases with a growing molar ratio of the TiO_2_ precursor. It was also observed that the peaks at 1630 cm^−1^ for O–H bending vibrations at 1400 cm^−1^ for C–O stretching vibrations decrease when the calcination temperature is increased.

### 3.6. Thermal Analysis

**Figure 9:**
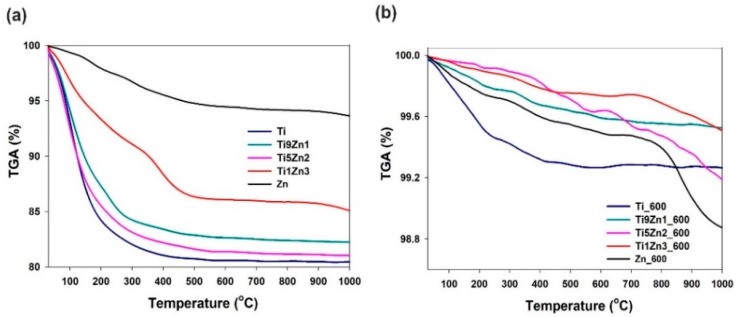
Thermal stability of TiO_2_, ZnO, and TiO_2_-ZnO oxide systems: (**a**) un-calcined and (**b**) calcined.

Analysis of the thermograms of samples Ti, Ti9Zn1, Ti5Zn2, and Zn (see Figure 9a) indicates a one-step degradation process. The degradation step in the temperature range 30 °C to 380 °C is associated with a significant decrease in mass by about 19%, 18.5%, 17.0%, and 2.5% for samples Ti, Ti5Zn2, Ti9Zn1, and Zn, respectively. The mass loss is mainly related to the local elimination of water bonded with the surface of the materials. When the temperature is above 380 °C, the samples stabilize and their mass remains almost unchanged. For sample Ti1Zn3, three mass losses were observed on the TGA curves. The first sample in the range of 30 °C to 300 °C corresponds to the loss of free water and amounts to about 7.5%. In the range 300°C to 470 °C, there is a second mass loss of about 5%, which is related to the thermal decomposition of the ZnO precursor. The total mass loss for sample Ti1Zn3 was 14.0%.

The thermograms of samples Ti_600 and Ti9Zn1_600 (see Figure 9b) show a minor mass loss in the temperature range of 30 °C to 350 °C by about 0.6% and 0.2%, respectively. This is related to the presence of a small amount of moisture in the systems [58,59]. In the range of 350 °C to 1000 °C, there is a second mass loss of about 0.7% and 0.4% for samples Ti_600 and Ti9Zn1_600, respectively. A slightly different thermogravimetric curve was observed for samples Ti5Zn2_600, Ti1Zn3_600, and Zn_600. In all three cases, the first degradation step in the temperature range of 30 °C to 300 °C with a mass loss of about 0.1% (for samples Ti5Zn2_600 and Ti1Zn3_600) and 0.2% (for sample Zn_600) is related to the local elimination of water bonded with the surface of the products. The next mass loss of about 0.2%, 0.4%, and 0.5% for samples Ti1Zn3_600, Ti5Zn2_600, and Zn_600, respectively, in the temperature range of 300 °C to 800 °C is related to the thermal decomposition of unreacted zinc acetate [60]. The third degradation step is probably associated with the phase transformation as a result of the applied high temperatures. The total mass loss for samples Ti5Zn2_600, Ti1Zn3_600, and Zn_600 is slightly above 0.8%, 0.3%, and 1.1%, respectively.

Wang et al. [61] who obtained nanoparticles of TiO_2_-ZnO via a sol-gel process observed three steps of mass loss, which are associated with the evaporation of water, the dehydroxylation of precursors, and the polymorphic transformation of TiO_2_. The results presented above indicate that the obtained TiO_2_-ZnO oxide systems have greater thermal stability than materials obtained in other studies [41,61]. In addition, titanium dioxide and zinc oxide following thermal treatment have similar thermal stability to what was reported in the literature [58,59,60,61].

### 3.7. Photocatalytic Activity

**Figure 10:**
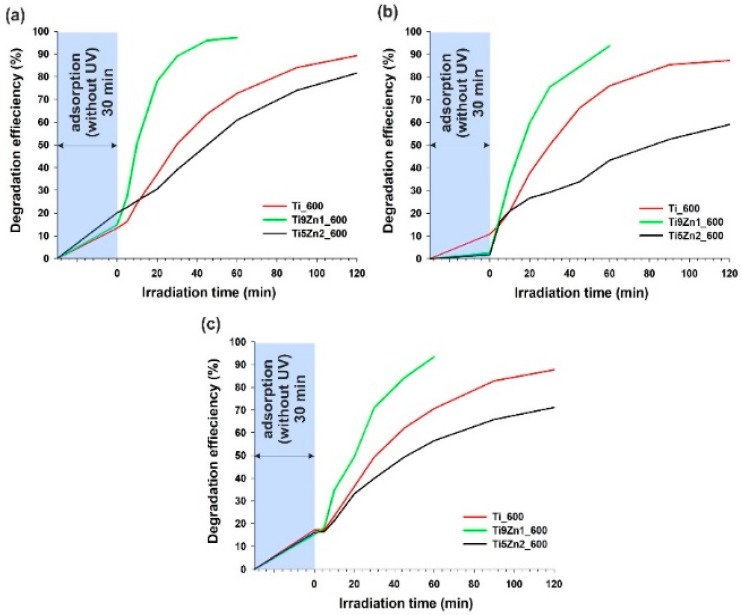
Efficiency of photocatalytic decomposition of (**a**) C.I. Basic Blue 9; (**b**) C.I. Basic Red 1; and (**c**) C.I. Basic Violet 10 in the presence of the synthesized hybrids.

Titanium dioxide is known as an effective photocatalyst for the photo-oxidation of different kinds of hazardous organic pollutants in waste water [15]. Zinc oxide is another attractive semiconductor oxide with similar photocatalytic properties [28]. For this reason, a key element of the present research was an evaluation of the photocatalytic activity of the obtained TiO_2_-ZnO binary oxide systems. The evaluation was based on the decomposition of MB, R6G, and RhB dyes under UV irradiation. Titanium dioxide and samples obtained with TiO_2_:ZnO molar ratios of 9:1 and 5:2, additionally calcined at 600 °C, were subjected to photocatalytic tests. The results are presented in Figure 10.

The first stage of photocatalytic testing involved evaluating the photocatalytic activity of TiO_2_-ZnO oxide systems in the removal of C.I. Basic Blue 9 (see Figure 10a). The TiO_2_-ZnO sample obtained with a molar ratio of TiO_2_:ZnO = 9:1 exhibited significantly better photocatalytic activity than pure titanium. Applying the Ti9Zn1_600 photocatalyst, the degree of decomposition of MB dye was 97.2% after 60 min of UV irradiation. The efficiency of C.I. Basic Blue 9 photodegradation in the presence of samples Ti_600 and Ti5Zn2_600 was 89.3% and 81.6% (after 120 min), respectively. Additionally, the photodecomposition of this organic dye increased with increasing irradiation time.

The decolorization of R6G under UV irradiation showed that sample Ti9Zn1_600 had good photo-oxidation activity (the efficiency of its degradation of C.I. Basic Red 1 was 93.6% after 60 min), which is shown in Figure 10b. Samples Ti_600 and Ti5Zn2_600 showed lower photocatalytic activity in the decomposition of C.I. Basic Red 1. The degradation efficiency was 87.2% (after 120 min) in the presence of sample Ti_600 and slightly lower (59.1%) in the case of photocatalysis using the sample Ti5Zn2_600.

Lastly, the photocatalytic experiments showed that a combination of titania with zinc oxide in a molar ratio of 9:1 exhibited significantly better photocatalytic activity than samples Ti_600 and Ti5Zn2_600 in the degradation of RhB (see Figure 10c). After 60 min of UV irradiation applying the Ti9Zn1_600 photocatalyst, the degree of decomposition of C.I. Basic Violet 10 reached 93.4%. The efficiency of degradation of RB dye in the presence of samples Ti_600 and Ti5Zn2_600 was 87.7% and 71.1%, respectively.

**Figure 11:**
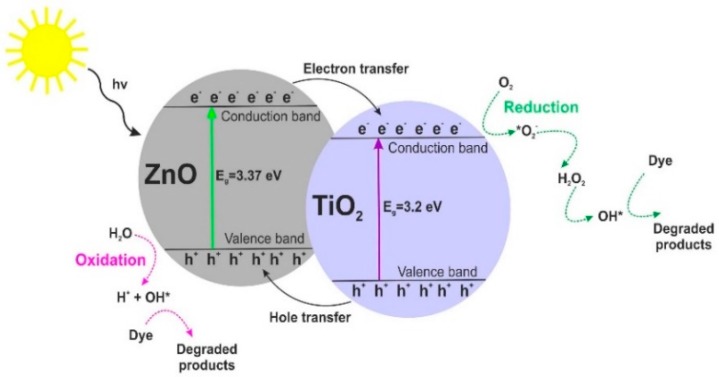
Mechanism of photodegradation of organic dyes using TiO_2_-ZnO oxide materials.

Our results imply that the photocatalytic activity of the synthesized samples depends not only on their BET surface area or crystallinity but can rather be attributed to dispersion and surface morphology. Moreover, based on research reports regarding heterogeneous photocatalysis [31,62,63], we propose a probable mechanism (see Figure 11) and reactions of the photodegradation of organic dyes using TiO_2_-ZnO oxide materials.

(5) $$\begin{array}{cc} TiO_{2}-ZnO & photocatalysts \end{array}+hv\to e^{-}+h^{+}$$

(6) $$h^{+}+H_{2}O\to H^{+}+OH^{*}$$

(7) $$h^{+}+OH^{-}\to OH^{*}$$

(8) $$h^{+}+dye\to \begin{array}{cc} oxidation & products \end{array}$$

(9) $$e^{-}+O_{2}\to {}^{*}O_{2}^{-}$$

(10) $${}^{*}O_{2}^{-}+2H^{+}\to H_{2}O_{2}$$

(11) $$e^{-}+H_{2}O_{2}\to OH^{*}+OH^{-}$$

(12) $$e^{-}+dye\to \begin{array}{cc} reduction & products \end{array}$$

(13) dye+OH*+O→2CO2+H2O+otherdecompositionproducts

Prasannalakshmi and Shanmugam [51] reported that TiO_2_-ZnO oxide hybrids obtained using a sol-gel method produce almost complete degradation of C.I. Basic Blue 9 within 25 min of irradiation. Pérez-González et al. [45] obtained (TiO_2_)_1−x_-(ZnO)_x_ thin films, with x = 0.00, 0.25, 0.50, 0.75, and 1.00, by the sol-gel process, which were deposited on glass. The synthesized films were evaluated for their ability to degrade MB. The authors found that the photocatalytic performance was improved by decreasing the value of x with the TiO_2_ thin films displaying the highest response. Araújo et al. [64] produced TiO_2_-ZnO hierarchical hetero nanostructures following a two-step procedure in which the hydrothermal growth of nanorods took place on the surface of decorated electrospun fibers. The resulting material was applied as a photocatalyst in the photodegradation of Rhodamine B. Photocatalytic tests showed the TiO_2_-ZnO composite to have good photocatalytic activity. Agrawal et al. [65] obtained hierarchically nanostructured hollow spheres composed of ZnO-TiO_2_ mixed oxides as a potential candidate for photocatalytic application. Pei and Leung [62] prepared TiO_2_-ZnO nanofibers from a nozzle-less electrospinning solution system. The authors evaluated the photocatalytic activities of different TiO_2_-ZnO composites in the photodegradation of Rhodamine B (RhB) under irradiation with 420 nm visible light. ZnO/TiO_2_ hybrid nanofibers were prepared via electrospinning by Chen et al. [63]. Based on the photodegradation of RhB, it was shown that the synthesized products exhibited high degradation efficiency. The ZnO/TiO_2_ (1 wt %) nanofibers degraded 90% of the dye in about 15 min.

Table 4 presents results from the literature concerning the efficiency of decomposition of C.I. Basic Blue 9, C.I. Basic Red 1, and C.I. Basic Violet 10 dyes when different photocatalysts were used.

Analysis of the kinetics of photochemical decomposition of organic dyes shows significant differences in the rate of degradation of the analyzed impurities in the presence of catalysts (see Table 5). Regardless of the type of organic dye, the highest values of the degradation reaction rate k (0.0596 min^−1^—C.I. Basic Blue 9, 0.0459 min^−1^—C.I. Basic Red 1 and 0.0453 min^−1^—C.I. Basic Violet 10) were recorded when the Ti9Zn1_600 oxide system was used as a photocatalyst. Furthermore, in the presence of Ti9Zn1_600 material, the highest values of the half-life time (*t*_1/2_ = 11.632 min—C.I. Basic Blue 9, 15.086 min—C.I. Basic Red 1 and 15.301 min—C.I. Basic Violet 10) of tested organic dyes were noted.

## 4. Conclusions

The proposed methodology of synthesis of the TiO_2_-ZnO binary oxide materials using the sol-gel method proved to be very effective. 

We studied how the TiO_2_:ZnO molar ratio and calcination temperature affects the physicochemical and photocatalytic properties of synthetic TiO_2_-ZnO oxide hybrids. It was found that the particle sizes, crystalline phase, surface area, pore structures, and photocatalytic activity of the TiO_2_-ZnO oxide systems are strongly dependent on the amount of zinc oxide in the product as well as on the calcination temperature.

The results of XRD analysis show that the quantity of zinc oxide in the product and the calcination temperature have significant effects on crystallizing the resulting materials. The porous structure parameters of the TiO_2_-ZnO oxide systems decreased with an increasing quantity of zinc oxide and temperature of calcination. 

The TiO_2_-ZnO oxide hybrid obtained in a molar ratio of TiO_2_:ZnO = 9:1 and calcined at 600 °C (sample T9Zn1_600) showed the highest photocatalytic activity. This is attributed to the fact that this sample is composed with titanium, zinc oxide, and ZnTiO_3_ phases as well as with anatase as the dominant phase. Moreover, analysis of the kinetics of the photocatalytic process performed based on the Langmuir-Hinshelwood equation confirmed that degradation of the model organic dyes occurred most intensely in the presence of the Ti9Zn1_600 catalyst.

## Figures and Tables

**Table 1:** Organic dyes used in photocatalytic tests.

Color Index Name	C.I. Basic Blue 9	C.I. Basic Red 1	C.I. Basic Violet 10
Chemical structure	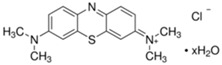	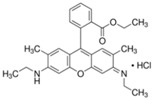	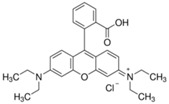
Molecular formula	C_16_H_18_ClN_3_S·3H_2_O	C_28_H_31_ClN_2_O_3_	C_28_H_31_ClN_2_O_3_
Molar mass (g/mol)	373.85	479.01	479.01
*λ_max_* (nm)	664	526	553

**Table 2:** Dispersive properties of TiO_2_-ZnO oxide systems obtained via the sol-gel method.

Sample Name	Molar Ratio TiO_2_:ZnO	Temperature of Calcination (°C)	Particle Diameter Range (nm)	Dominant Particles Diameter with Maximum Volume Contribution (%)	Polydispersity Index
Ti	10:0	-	531–1720	955 nm—21.6	0.183
Ti9Zn1	9:1		459–1110	712 nm—27.5	0.312
Ti5Zn2	5:2		459–1480	825 nm—25.9	0.361
Ti1Zn3	1:3		396–825	615 nm—31.7	0.150
Zn	0:10		220–615	396 nm—25.7	0.178
Ti_600	10:0	600	615–1990	1110 nm—23.8	0.220
Ti9Zn1_600	9:1		531–1280	825 nm—32.6	0.408
Ti5Zn2_600	5:2		459–955	712 nm—32.2	0.504
Ti1Zn3_600	1:3		255–615	396 nm—26.7	0.182
Zn_600	0:10		51–122 220–1110	79 nm—5.1 531 nm—14.2	0.434

**Table 3:** Porous structure parameters of TiO_2_-ZnO oxide systems obtained by the sol-gel method.

Sample Name	Molar Ratio TiO_2_:ZnO	Temperature of Calcination (°C)	Specific Surface Area A_BET_ (m^2^/g)	Total Pore Volume V_p_ (cm^3^/g)	Average Pore Size S_p_ (nm)
Ti	10:0	-	488.6	0.046	1.9
Ti9Zn1	9:1		494.7	0.079	1.9
Ti5Zn2	5:2		475.8	0.051	1.9
Ti1Zn3	1:3		97.0	0.030	2.0
Zn	0:10		27.2	0.008	2.1
Ti_600	10:0	600	26.5	0.010	2.2
Ti9Zn1_600	9:1		7.6	0.003	2.1
Ti5Zn2_600	5:2		7.2	0.005	2.2
Ti1Zn3_600	1:3		7.5	0.005	2.3
Zn_600	0:10		11.5	0.007	2.2

**Table 4:** Efficiency of decomposition of selected organic dyes.

Sample Name	Concentration of Dye Solution (mg/dm^3^)	Efficiency of Decomposition (%)			Ref.
		C.I. Basic Blue 9	C.I. Basic Red 1	C.I. Basic Violet 10	
Ti9Zn1_600	5	97.2	93.6	93.4	this work
(TiO_2_)_1−x_-(ZnO)_x_	10	45.0–62.0	-	-	[45]
TZO1-TZO4	1	~100	-	-	[51]
TiO_2_	5	98.3	-	-	[59]
TiO_2_/ZnO	0.5	-	-	~100	[62]
ZnO/TiO_2_	2	-	-	90.0	[63]
TiO_2_/ZnO	4.8	-	-	90.0	[64]
TiO_2_/ZnO	4.8	-	~100	-	[65]
ZnO-TiO_2_	4.8	-	-	~100	[66]
TiO_2_/ZnO	20	96.0	-	83.0	[67]

**Table 5:** The reaction rate constant (*k*), correlation coefficient (*R*^2^), and half-life time (*t*_1/2_) of tested organic dyes during the photocatalytic process.

Sample Name	*k* (1/min)	*R^2^*	*t*_1/2_ (min)
***C.I. Basic Blue 9***			
Ti_600	0.0186	0.9937	37.269
Ti9Zn1_600	0.0596	0.9731	11.632
Ti5Zn2_600	0.0141	0.9957	49.178
***C.I. Basic Red 1***			
Ti_600	0.0172	0.9584	40.398
Ti9Zn1_600	0.0459	0.9930	15.086
Ti5Zn2_600	0.0074	0.9922	93.225
***C.I. Basic Violet 10***			
Ti_600	0.0174	0.9918	39.727
Ti9Zn1_600	0.0453	0.9897	15.301
Ti5Zn2_600	0.0103	0.9765	67.007
